# Sex-Dependent Novelty Response in Neurexin-1α Mutant Mice

**DOI:** 10.1371/journal.pone.0031503

**Published:** 2012-02-13

**Authors:** Marijke C. Laarakker, Niels R. Reinders, Hilgo Bruining, Roel A. Ophoff, Martien J. H. Kas

**Affiliations:** 1 Department of Neurosciences & Pharmacology, Division of Neuroscience, Rudolf Magnus Institute, University Medical Center Utrecht, The Netherlands; 2 Department of Psychiatry, Division of Neuroscience, Rudolf Magnus Institute, University Medical Center Utrecht, The Netherlands; 3 Center for Neurobehavioral Genetics, University of California Los Angeles, Los Angeles, California, United States of America; Alexander Flemming Biomedical Sciences Research Center, Greece

## Abstract

Neurexin-1 alpha (NRXN1α) belongs to the family of cell adhesion molecules (CAMs), which are involved in the formation of neuronal networks and synapses. NRXN1α gene mutations have been identified in neuropsychiatric diseases including Schizophrenia (SCZ) and Autism Spectrum Disorder (ASD). In order to get a better understanding of the pleiotropic behavioral manifestations caused by NRXN1α gene mutations, we performed a behavioral study of Nrxn1α heterozygous knock-out (+/−) mice and observed increased responsiveness to novelty and accelerated habituation to novel environments compared to wild type (+/+) litter-mates. However, this effect was mainly observed in male mice, strongly suggesting that gender-specific mechanisms play an important role in Nrxn1α-induced phenotypes.

## Introduction

Recent genetic studies have demonstrated that Neurexin-1 alpha (NRXN1α) is involved in schizophrenia (SCZ) and Autism Spectrum Disorder (ASD). Deleterious mutations disrupting the open reading frame of the gene are associated with increased risk for schizophrenia [1], [2] whereas point mutations in the coding sequence have been described for ASD [3], [4]. Genomic copy number variations (CNVs) in NRXN1α lead to decreased or strongly diminished NRXN1α mRNA expression levels [3], [5]–[8]. Nrxn1α is a neuronal cell-surface protein that facilitates synaptic connectivity between neurons [9]. However, there is a large variety of isoforms that may have anatomical, functional and neuronal activity dependent specificity [10]–[16]. Known presynaptic functions of NRXNs are facilitation of vesicle exocytosis a and synapse structuring [17]–[19]. NRXNs are classified as Cell Adhesion Molecules (CAMs). Missler and colleagues [20] showed that alpha neurexins modulate presynaptic neuron terminals by locally activating Ca2+ channels and, thereby, mediating synaptic vesicle exocytosis. Several studies implicate that malfunction of various synaptic vesicle release mechanisms may cause behavioral impairment related to object and social cognition and motor functions [21]–[23]. A behavioral study in mice has shown that Nrxn1α deficiency results in a variety of phenotypes that do not always have detrimental effects [24]. For example, mice with a homozygous (−/−) deletion of Nrxn1α spent more time on grooming, but also showed improved motor learning. Since haploinsuffiency of NRXN1α deletions in humans is associated with SCZ and ASD [1], [25] and both SCZ and ASD patients show impairments in familiarizing to novel situations related to information processing [26]–[28] we studied novelty responsiveness and habituation to novel environments in Nrxn1α heterozygous knock-out mice (+/−) and compared this phenotype in female and male mice as also substantial gender differences exist in the manifestation of human autistic and psychotic traits (for reviews, see [29], [30]).

## Materials and Methods

### Animals

#### Mice

Heterozygous Nrxn1α KO (+/−) and wild-type (+/+) C57BL6/SV129 (WT) mice were used (n = 10 per genotype, per gender). These mice were generated by crossing the heterozygous Nrxn1α knock-out [24] on a C57BL6/SV129 background with C57BL/6J mice, to create a F2 of 50% heterozygous Nrxn1α heterozygous KO (+/−) mice and 50% WT (+/+) mice on a C57BL6/SV129 mixed genetic background. The heterozygous KO (+/−) mice were compared to their wild type (+/+) littermates to minimize unwanted genetic variance between groups. To assess the genotype of the animals, PCR amplification was used on genomic mouse DNA from ear punches. The knock out and wild type allele were separately amplified in a standard two primer PCR (KO primer: GAGCGCGCGCGGCGGAGTTGTTGAC, WT primer: CGAGCCTCCCAACAGCGGTGGCGGGA, common primer: CTGATGGTACAGGGCAGTAGAGGACCA). In 35 cycles the products were amplified (KO band: 400 bp, WT band: 500 bp) and the reaction was analyzed on a 2% agarose gel containing ethidium bromide. In all experiments we used mice between age 2–4 months. The animals were housed under standard housing conditions at the Animal Laboratory (GDL, Gemeenschappelijk Dieren Laboratorium, Utrecht University, The Netherlands). Macrolon type II cages (22 cm×16 cm×14 cm, floor area 350 cm^2^) in a closed air ventilation system. Mice were housed on wood-chip bedding and tissues were available as cage enrichment. Animals were housed in groups of 2–4 per cage with light periods from 7:00–19:00. Food and water was available ad libitum.

#### Ethics

The protocol of the animal experiments was approved by the Animal Experiments Committee of the Academic Biomedical Center, Utrecht, The Netherlands. The Animal Experiments Committee based its decision on ‘De Wet op de Dierproeven’ (1996) and on the ‘Dierproevenbesluit’ (1996); both are available online (http://www.nca-nl.org/). Additionally, all animal experiments followed the ‘principles of laboratory animal care’ and refer to the ‘guidelines for the Care and Use of Mammals in Neuroscience and Behavioral Research’ (National Research Council 2003). The approval ID number which was given by the Dutch Ethics Committe (DEC) that reviewed the protocol is: 2009.I.10.080.

### Behavioral testing

#### Novelty responsiveness

Exploration behavior and habituation to a novel environment of the mice was assessed in an empty macrolon cage type IV (dimensions: 55 cm×33 cm×20 cm (with a total floor area of 1815 cm^2^) located in a ventilated flow cabin. All mice were tested 3 times for 5 minutes with an inter-trial interval of 60 minutes. Males and female mice were tested on different testing days using different test cages for males and females to exclude odor disturbances from different gender. The critical parameter measured was total distance moved (representing non-specific horizontal motor activity levels). Behavioral scoring was performed automatically on video recordings using behavioral analysis software (EthoVision version 3.1, Noldus Information Technology Bv, Wageningen, Netherlands).

#### Object Discrimination

Long-term (24 hrs) object discrimination was performed in the same cage types as described above (macrolon cage type IV); this test was performed one week after testing for novelty responsiveness (as described above). For adaptation purposes two equal objects were paced into the test arena and the mice were allowed to freely explore the objects for 10 minutes. After 24 hours this test was repeated, however, one of the two objects was replaced by another novel (unfamiliar) object and mice were allowed to freely explore both objects again for 10 minutes. Three different equal sized objects (metal circular tin, blue with white dots; cone-shaped glass with green lines; square plastic box) were used and randomly chosen for each of the tested mice. Prior to these experiments, an other group of mice (n = 6 male WT mice and heterozygous KO (+/−) mice) was exposed to the objects used in this test and showed that mice had no natural preference for the selected objects (data not shown). Behavioral scoring was performed manually using software for behavioral testing (The Observer version 5.0, Noldus Information Technology Bv, Wageningen, Netherlands).

### Statistical analysis

All statistical analysis were performed in SPSS for Windows and carried out two-sided for all described tests. All continuous data were described by means and standard error of the mean (SEM). Normality of the data was checked by one-sample Kolmogorov-Smirnov test.

#### Novelty responsiveness

Normal distribution was revealed for all experimental groups for the parameters ‘total distance moved (cm)’ in the empty Macrolon cage and for the parameters describing the activity difference between trial 1 and 3. During analysis of the novelty responsiveness data the cage floor was divided into two zones, only visible to the analysis program but not physically present in the test set-up. This was determined by a smaller rectangle 10 cm from the sidewalls towards the middle of the set-up. The zone along the sidewalls is now referred to as ‘outer zone’ and the inner part of the set-up is referred to as ‘center zone’ [31], [32]. This difference was calculated for ‘total distance moved in the inner zone (cm)’ and ‘total time spent in the center zone (s)’. Following, homoscedacity was analyzed by the Levene's test. This criterion was only fulfilled for the parameter ‘total distance moved (cm)’ after log-transformation. A repeated measured ANOVA was performed on the log-transformed parameter ‘total distance moved’ using the Huynh-Feldt adaptation. Fixed factors in this model were genotype (heterozygous KO (+/−) or WT (+/+)), gender (male or female), and trial (1st, 2nd, or 3rd). Post-hoc analysis was carried out on factors revealing significance in the repeated measures ANOVA (alpha = 0.050). Post-hoc analysis was done by paired samples Student's t-test, where alpha was adapted to 0.0167 by Bonferroni-correction to correct for type-I errors due to multiple testing. The parameter describing the behavior in the center zone of the Macrolon cage (the difference between trial 1 and 3 for total distance moved and total time moving) was analyzed using an independent-samples Student's t-test. For each gender a genotype-comparison was performed.

#### Object Discrimination

In order to analyze object discrimination data a discrimination coefficient (f (t_exploration_)) was calculated. This coefficient describes the time spend exploring the novel object as a fraction of the total object exploration time:
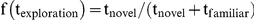
After analysis of the total testing time, the experimental time was divided into two 5-minute time bins and analyzed separately. Normal distribution was revealed for all experimental groups and discrimination coefficients where compared to the coincidence threshold level of 0.5 by one-sample t-test. Further, the two 5-minute time bins were compared between wild type and heterozygous mice by means of a paired samples t-test.

## Results

### Novelty responsiveness

**Figure 1:**
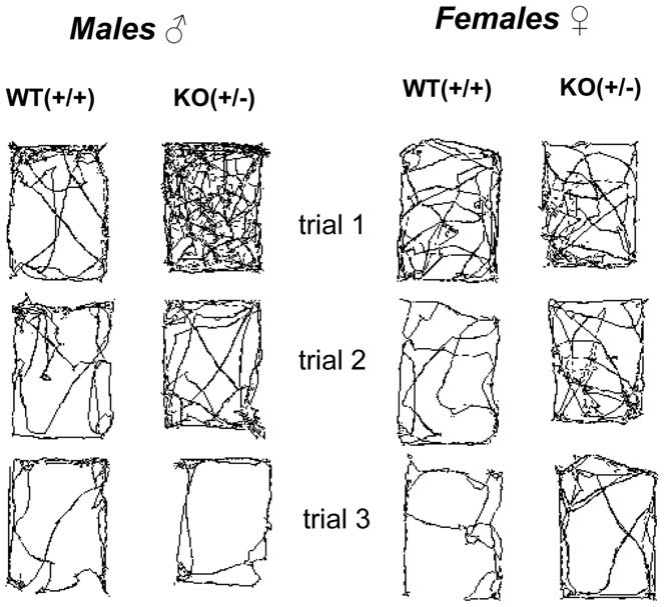
Representation of open field movement tracking pattern measured in individual male and female WT(+/+) and heterozygous KO(+/−) mice in three consecutive 5 minute trials.

All mice were allowed to explore the novel cage arena for 3 times (5 minutes per trail) with an inter-trial interval of 60 minutes (see Figure 1 for movement patterns in the novel Macrolon cage). A repeated measures analysis of variance for distance moved was performed with trial, gender and genotype as fixed factors. Analysis of between-genotype effects revealed a significant interaction of trial, gender and genotype reaching a p-value of 0.024. Due to the significant three-way interaction, the data were also analyzed per gender. Interestingly, this analysis revealed a significant genotype-trial interaction in the males (p = 0.023), but not in the females (p = 0.594), further indicating that male heterozygous KO (+/−) mice exhibited a stronger habituation response to repeated exposures to a novel environment when compared to their WT controls. In addition, the factor trial revealed a significant p-value (p<0.001).

**Figure 2:**
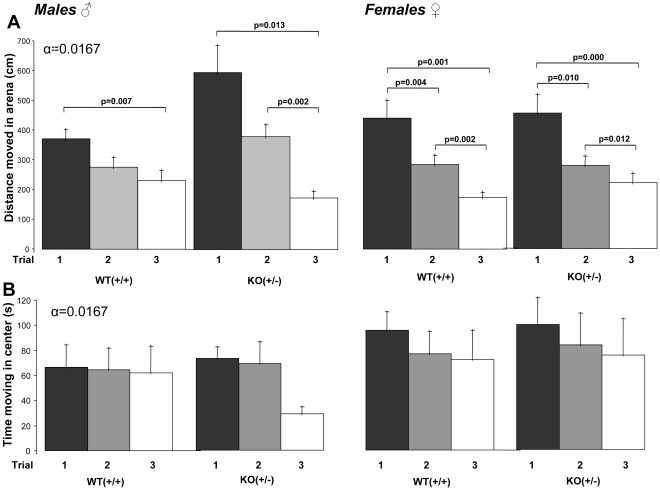
Total distance moved in the open field arena in cm (A) and total time spend in the center of the open field in seconds (B) were measured in the open field test in male and female heterozygous KO(+/−) and WT(+/+) mice (mean +/− SEM). Alpha was set at 0.0167 after Bonferroni correction.

Post-hoc analysis of the within-genotype effects showed that male heterozygous KO (+/−) mice explored the open field arena (i.e. total distance moved in the arena) mostly in the first trial, and showed a significant decrease of the overall distance moved with each subsequent trial (trial 1–3, p = 0.013; trial 2–3, p = 0.002). The total distance moved by WT male mice in the first trial was substantially less than that for the heterozygous KO (+/−) mice. In the WT mice the total distance moved in trial 1 was only significantly increased compared to trial 3 (p = 0.007) (Figure 2A; left panel). Post-hoc analysis for the females showed significant trial differences for both, WT and heterozygous KO (+/−) mice between all trials (heterozygous KO (+/−): trial 1–2, p = 0.010; trial 2–3, p = 0.012; trial 1–3, p = 0.001 and WT: trial 1–2, p = 0.004; trial 2–3, p = 0.002; trial 1–3, p = 0.001) (figure 2A; right panel). Repeated measures analysis of variance for time spent in the center was performed with trial, gender and genotype as fixed factors, however, no interaction effect was observed (figure 2B).

**Figure 3:**
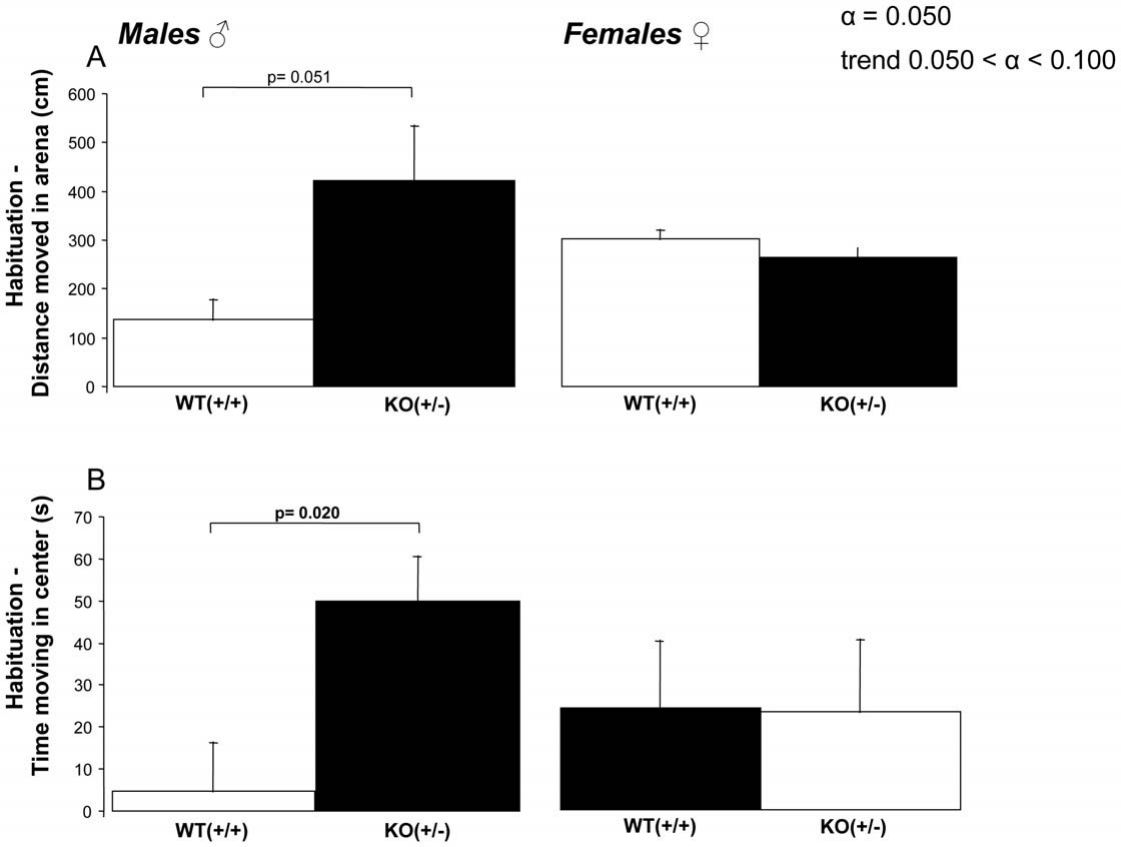
Habituation was described by the difference between trial 3 and trial 1, determined for the total distance moved in the open field (A) and the total time spend in the center of the open field test (B). Error bars are standard errors of the mean. P-values of less than 0.050 shows significant effects, while p-values between 0.050 and 0.100 indicate a trend.

In order to analyze habituation behavior on the basis of multiple exposures to the cage environment, we examined the difference between the levels of distance moved during the first and the last trials for each experimental group, as well as the total time spent in the center zone of the cage (Figure 3). Habituation was defined by the difference between trial 1 and 3 (Δ trial 1–3). This analysis revealed that male heterozygous KO (+/−) mice differed significantly in habituation to the novel environment when compared to WT mice. Genotype differences for male mice were found for the trial difference in total distance moved (p = 0.051) and for trial difference in total time spent in the center (p = 0.020) (figure 3A and B; left panels). In contrast to the male mice, the level of habituation of female WT and heterozygous KO (+/−) mice to the novel environment was not significantly different (p>0.05). For females, the differences between trial 1 and 3 for the distance moved in the center and the total time spent in the center did not show any genotype differences (p = 0.622 an p = 0.962, respectively) (figures 3A and 3B; right panels).

### Object Discrimination

To study the consequences of the genotype effects of enhanced habituation in male mice, WT and heterozygous KO (+/−) males were also tested for object discrimination strategies. Male mice were tested 24 hrs after adaptation to the familiar objects for object discrimination capacity between a familiar and novel object. In order to assess possible genotype effects on the rate of discrimination capacity, discrimination coefficients were calculated for the total 10 minute trial, and for the first and second 5 minute time bin of the total testing time.

**Figure 4:**
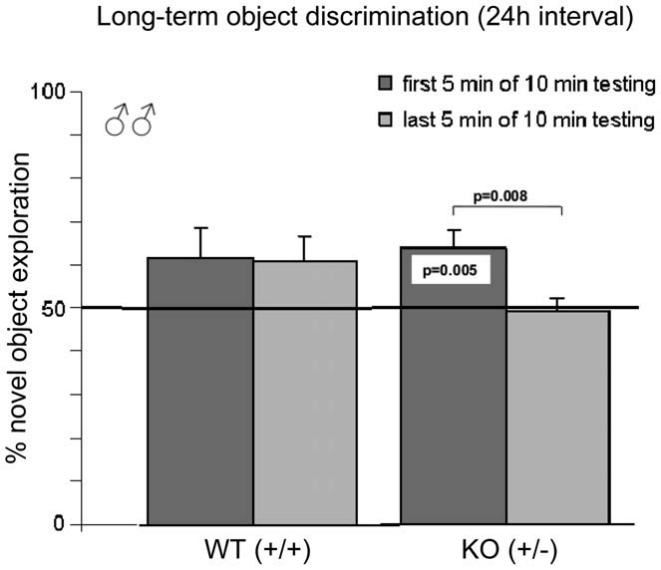
Object discrimination was measured by the relative time male mice spend exploring the novel (unknown) object. Significant longer exploration of the novel object compared to the 50% coincidence level indicates recognition of the novel object compared to the familiar one (group mean +/− SEM).

The analysis showed that WT mice showed a significant discrimination capacity between the novel and familiar objects over the 10 minute trial (p = 0.010). In contrast, heterozygous KO (+/−) mice seemed to lack object discrimination capacity, as they show similar exploration time to the familiar and novel object during a 10 minute testing trial (p = 0.070), indicating that the heterozygous KO (+/−) mice are less capable to discriminate. However, given by the enhanced habituation time in the open field, we wondered whether heterozygous KO (+/−) mice are capable to discriminate between a familiar and novel object at a faster rate compared to WT mice. Consistent with this notion, in the first 5 minutes of exploration of the objects, male heterozygous KO (+/−) mice showed significant discrimination capacity and thus, higher novel object exploration time (p = 0.005), in contrast to the subsequent 5 minute bin (Figure 4).

## Discussion

This study was designed to examine the effect of haploinsufficiency of Nrxn1α on murine behavior given the fact that microdeletions in the human orthologous gene are associated with SCZ while point mutations are linked with ASD. Nrxn1α heterozygous KO (+/−) mice showed increased locomotor activity levels in a new environment and enhanced habituation upon subsequent exposures to this environment. Interestingly, genotype differences in novelty responsiveness were only observed in male mice, indicating sex-specific differences of the behavioral phenotype as a function of the Nrxn1α heterozygous deletion. Thus, a difficulty to change or to cope with novel situations might be more pronounced in male carriers of NRXN1 mutations in humans as well. This observation suggests that an altered novelty response in Nrxn1 mutant mice may be a translational phenotype for ASD and SCZ given the male preponderance for these disorders. Gender bias in novelty response in mice has been reported before with enhanced exploration in males [33], [34]. However, our results show that haploinsufficiency of Nrxn1a affects novelty response in males disproportionally compared to females. Larger study samples are needed to fully decipher the extent of the observed gender differences and whether genetic background plays a role.

In addition to the levels of movement in the novel cage environment, the time spent in the center of the cage is commonly measured and thought to reveal anxiety-related behavior in rodents. In the present study, wild type male, as well as wild type and heterozygous KO (+/−) female mice spent equal time in the center, with no differences between trials. The locomotor phenotype in the novel cage environment was not reported in the initial behavioral study in Nrxn1α deficient mice [24]. This may be due to the differences in genotypes studied (heterozygous (this study) or homozygous gene knockouts) or due to the separate analysis of female and male data in the present study. The latter would be supported by the notion that combining our female and male novelty responsiveness data would also reveal no behavioral phenotype for mice with this gene deletion.

Behavioral effects of Nrxn1α deletion in the current male genetic background seem to exert effects in the domain of novelty exploration. In addition, male heterozygous KO (+/−) mice also showed a more rapid novel object discrimination capacity. These findings indicate that heterozygous KO (+/−) and WT mice both discriminate successfully between a familiar and novel object within a 10 minute trial, but that the male KO mice discriminate at a faster rate compared to the WT mice (within 5 minutes versus 10 minutes, respectively). This observation suggests that this genetic deletion in Nrxn1α may enhance learning and memory processes that are related to novel objects. These findings seem consistent with a previous study revealing enhanced motor learning capacity in Nrxn1α deficient mice when these mice were studied on the rotarod [24]. It is unknown how enhanced motor learning may relate to increased grooming behavior that has been observed in Nrxn1α deficient mice [24]. Further studies are needed to understand how Nrxn1α function in motor learning can be linked to the molecular function of Nrxn1α. Neurexins act as synaptic bridge to ensure structural integrity and functioning of the vesicle release apparatus. Therefore heterozygous Nrxn1α deletion could reduce this integrity and reduce the chance of vesicle release [16], [20], [35]. This will affect the pruning dynamics and could lead to faster pruning of the affected synapses [36] and related learning processes. While further studies are necessary to proof this hypothesis, current and previous findings [15], [37], [38] suggest that Nrxn1α has, through its effects on synaptic regulation, an important contribution to relevant behavior related to adaptation to new situations, particularly in males.
